# Celebrating novel cancer drugs

**DOI:** 10.1038/sj.bjc.6601229

**Published:** 2003-07-29

**Authors:** Robin A Weiss

In this issue, we publish an unusual Meeting Report (p 437). It looks back over 20 years of drug development inspired by the late Tom Connors and brought to fruition through the work of the Phase I/II Clinical Trials Committee of Cancer Research UK (formerly of the Cancer Research Campaign). In celebrating impressive achievements and current strengths, the speakers also look forward to further progress and development ranging from small molecular weight antagonists of cell proliferation and endocrine stimulation to antibody-targeted treatment and gene therapy based on the metabolic activation of prodrugs.

What is remarkable is that this Clinical Trials Committee in the UK has selected some 89 agents for 85 Phase I trials, and 30 have progressed to further clinical investigation. The Committee had a small budget but large ambitions. It has won great respect not only in the British community of clinical researchers, but also at the US National Cancer Institute and from the European Organisation for Research and Treatment of Cancer (EORTC). Tom Connors, a trusted collaborator and facilitator, tirelessly built up a network of formal and informal links that led to many international successes. Drugs such as temozolomide, 4-hydroxyandrostenedione and carboplatin were first tested in patients under the auspices of the Phase I/II Clinical Trials Committee.

Phase I and II clinical trials, carefully supervised and conducted to the highest standards, are crucial before investing in Phase III randomised control trials involving large numbers of patients and considerable costs. For every success in this field, there are many near misses and failures, so it is also important both to patients and the pharmaceutical industry to know when to abandon a drug that initially appeared promising. The Phase I/II Clinical Trials Committee has never shied away from harsh decisions over its members' favourite therapeutic. It has insisted on the highest standards in accordance with the requirements of international regulatory agencies when evaluating potential therapies, whilst at the same time supporting the development of novel means of measuring the efficacy, pharmacokinetics and pharmacodynamics of the drugs in action.

As the Meeting Report concludes, by any standards the achievements of the Phase I/II Committee have made an impressive contribution to international anticancer drug discovery and evaluation, to the benefit of cancer patients. That is due in no small part to the vision, enthusiasm and rigour of its founders. Those who now constitute the Committee are well placed to continue its role in introducing future drugs based on rational design tailored to targets revealed by our growing understanding of the molecular pathways that become aberrant in cancer cells.

